# Sodium–Glucose cotransporter 2 inhibitor empagliflozin decreases ventricular arrhythmia susceptibility by alleviating electrophysiological remodeling post-myocardial-infarction in mice

**DOI:** 10.3389/fphar.2022.988408

**Published:** 2022-10-14

**Authors:** Genlong Xue, Xiaolei Yang, Ge Zhan, Xin Wang, Jinghan Gao, Yong Zhao, Xinying Wang, Jiatian Li, Zhenwei Pan, Yunlong Xia

**Affiliations:** ^1^ Institute of Cardiovascular Diseases, The First Affiliated Hospital of Dalian Medical University, Dalian, China; ^2^ Department of Cardiology, The First Affiliated Hospital of Dalian Medical University, Dalian, China; ^3^ Department of Ultrasound, The Affiliated Hospital of Innermongolia Medical University, Huhhot, China; ^4^ Department of Pharmacology (The Key Laboratory of Cardiovascular Research, Ministry of Education) at College of Pharmacy, Harbin Medical University, Harbin, China

**Keywords:** sodium–glucose cotransporter 2, empagliflozin, myocardial infarction, membrane potential, intracellular Ca^2+^

## Abstract

**Background:** Recent clinical trials indicate that sodium–glucose cotransporter 2 (SGLT2) inhibitors improve cardiovascular outcomes in myocardial infarction (MI) patients, but the underlying mechanisms remain unknown. As arrhythmia often occurs during myocardial infarction, it is the main cause of death.

**Objective:** The purpose of this study was to investigate the influence of empagliflozin (EMPA), an SGLT2 inhibitor, on cardiac electrophysiological remodeling and arrhythmia susceptibility of myocardial infarction mice.

**Methods:** ECG was obtained from mice 1 week after MI to determine the QT interval. In an electrophysiological study and optical mapping was performed to evaluate the function of EMPA and underlying mechanisms of post-myocardial-infarction in mice.

**Results:** EMPA treatment significantly reduced the QT interval of MI mice (MI + EMPA 50.24 ms vs. MI 64.68 ms). The membrane potential and intracellular Ca [Ca_i_] were mapped from 13 MI hearts and five normal hearts using an optical mapping technique. A dynamic pacing protocol was used to determine action potential duration and [Ca_i_] at baseline and after EMPA (10 umol/L) infusion. EMPA perfusion did not change the APD_80_ and CaT_80_ in normal ventricles while shortening them in an infarct zone, bordering zone, and remote zone of MI hearts at 200 ms, 150 ms, 120 ms, and 100 ms pacing cycle length. The conduction velocity of infarcted ventricles was 0.278 m/s and 0.533 m/s in normal ventricles at baseline (*p* < 0.05). After EMPA administration, the conduction velocity of infarcted ventricles increased to 0.363 m/s, whereas no significant changes were observed in normal ventricles. The action potential rise time, CaT rise time, and CaT tau time were improved after EMPA perfusion in infarcted ventricles, whereas no significant changes were observed in normal ventricles. EMPA decreases early afterdepolarizations premature ventricular beats, and ventricular fibrillation (VF) in infarcted ventricles. The number of phase singularities (baseline versus EMPA, 6.26 versus 3.25), dominant frequency (20.52 versus 10.675 Hz), and ventricular fibrillation duration (1.072 versus 0.361 s) during ventricular fibrillation in infarcted ventricles were all significantly decreased by EMPA.

**Conclusion:** Treatment with EMPA improved post-MI electrophysiological remodeling and decreased substrate for VF of MI mice. The inhibitors of SGLT2 may be a new class of agents for the prevention of ventricle arrhythmia after chronic MI.

## Introduction

Ventricular arrhythmias are the main cause of mortality in patients suffering from myocardial infarction (MI) (Behnes et al., 2018; Frontera et al., 2020). Myocardial infarction is associated with significant electrophysiological remodeling including aberrant cardiac conduction and altered action potential duration (APD) in post-MI hearts (Jiang et al., 2007; Mendonca Costa et al., 2018; Chowdhury et al., 2021). The inhomogeneous prolongation of APD and slowing of cardiac conduction velocity in the infarct zone (IZ), bordering zone (BZ), and remote zone (RZ) are prone to induce arrhythmias in infarcted hearts (Mendonca Costa et al., 2018; Wen et al., 2018). Post-MI malignant ventricular arrhythmias were also related to abnormal Ca^2+^ handling (Belevych et al., 2012). The functional disorder of calcium transient dynamics leads to Ca^2+^ cycling disturbance in infarcted ventricles, which induces secondary rises of [Ca_i_] and triggers early afterdepolarizations (EADs) and ventricular arrhythmias (Chang et al., 2013). Irregularity of APD and calcium transient has been taken as a hallmark of cardiac electrical disturbances after MI.

Sodium–glucose cotransporter 2 (SGLT2) inhibitors such as empagliflozin (EMPA) comprise a group of anti-hyperglycemic drugs that promote urinary excretion of glucose by inhibiting glucose reabsorption through SGLT2 blockade (Kondo et al., 2021). Surprisingly, recent large-scale cardiovascular safety trials demonstrate that SGLT2 inhibitors exert not only antidiabetic actions but also cardioprotective effects (Zelniker and Braunwald, 2020)and the use of SGLT2 inhibitors in patients with diabetes mellitus (DM), chronic kidney disease (CKD), and heart failure (HF) to reduce related cardiac complications and comorbidities of cardiac arrhythmias (Li et al., 2021). Clinical research findings showed that dapagliflozin proved to be clinically effective in patients with heart failure (HF) with reduced ejection fraction regardless of diabetes, suggesting its robust benefits in some specific patients with HF (Tanaka and Node, 2020). Kondo et al. (2021) discovered that canagliflozin suppressed myocardial NADPH oxidase activity and improved NOS coupling to anti-inflammatory and ant-apoptotic effects in the human myocardium, indicating that the cardioprotective effect was not related to their systemic glucose-lowering function.

SGLT2 inhibitor, empagliflozin, was shown to attenuate cardiac fibrosis (Li et al., 2019a) and pressure overload-induced heart failure (Yurista et al., 2019). Lately, Philippaert et al. (2021) demonstrated that empagliflozin inhibits late-*I*_Na_ in a transaortic constriction (TAC)-induced mouse heart failure model, prevents the activation of the NLRP3 inflammasome, and improves functional recovery in an *ex vivo* cardiac ischemia/reperfusion injury model. However, the action of empagliflozin on electrophysiological remodeling and arrhythmia susceptibility during chronic myocardial infarction remains unclear.

Therefore, in this study, we explored the effects of empagliflozin on action potential repolarization and dynamics of calcium transient in chronic infarcted ventricles of mice. We found that empagliflozin reduced ventricular arrhythmia susceptibility by shortening APD_80_ and CaT_80_ of infarcted ventricles, improving the dynamics of AP rise time, CaT rise time, and Tau and decreasing the occurrences of EADs, PVBs, and secondary rises of [Ca_i_]. The study highlights a novel mechanism for the regulation of empagliflozin on ventricular arrhythmia and reveals its therapeutic potential for post-MI-related cardiac arrhythmias.

## Methods

### Animal studies

This study was approved and monitored by the Laboratory Animal Resource Center at Dalian Medical University and conformed to the Guide for the care and use of laboratory animals published by the US National Institutes of Health (NIH Publication No. 85–23, revised 1996). Adult C57BL/6 mice (8 weeks old) were provided by the animal center at Liaoning Changsheng Biotechnology Co., Ltd.

### Myocardial infarction model

C57BL/6 mice underwent MI, as described previously (Alibhai et al., 2014). Mice were anesthetized by spontaneous inhalation and maintained under general anesthesia with 2% isoflurane. Animals were intubated and ventilated with air using a small-animal respirator. The heart was exposed by a left-side limited thoracotomy, and the left ventricle was visualized and the pericardial sac was ruptured to expose the left anterior descending (LAD) coronary artery. The LAD was ligated with a 7–0 prolene suture 2 mm below the tip of the left auricle. We studied the EMPA (10 umol) impact on MI hearts that followed up 1 week after MI.

### Electrophysiological study

Mice were anesthetized as previously described (Si et al., 2020). Intervals (HR, QRS, QT, and RR) were measured and analyzed using a BL-420 Biological Signal Acquisition System (Chengdu Techman Software Co. Ltd., Chengdu, China). Surface electrocardiograph (ECG) was recorded. QTc interval was calculated as QT interval (ms) divided by the square root of the RR interval (ms) divided by 100 (Mitchell et al., 1998). The maximum slope-–intercept method was used to define the end of the T-wave.

### Optical mapping

Mice were heparinized and euthanized with 2,2,2-tribromoethanol (200 mg/kg, intraperitoneal injection; Sigma, St Louis, MO, United States). The heart was isolated and Langendorff perfused with Tyrode’s solution (NaCl 128.2 mM, CaCl_2_•2H_2_O 1.3 mM, KCl 4.7 mM, MgCl_2_•6H_2_O 1.85 mM, NaH_2_PO_4_•2H_2_O 1.19 mM, Na_2_CO3 20 mM, and glucose 11.1 mM; pH 7.35) at 37°C. After 10 min of stabilization, the hearts were stained with Rhod-2 AM (1.48 μM) for [Ca_i_] and RH237 (10 μM) for membrane voltage (Vm) mapping. The double-stained hearts were excited at 710 nm wavelength for Vm and 580 nm wavelength for [Ca_i_] using a two-wavelength light-emitting device. The fluorescence was filtered and recorded simultaneously with an OMS-PCIE-2002 camera (MappingLab, United Kingdom) at 1 ms/frame and 100 × 100 pixels with a spatial resolution of 0.35 × 0.35 mm^2^ per pixel. Optical signals were processed with both spatial (3 × 3 pixels Gaussian filter). Phase mapping was performed to evaluate the location and evolution of phase singularities (PSs). Blebbistatin (10–20 μmol/L, Tocris, Ellisville, and MO) was used to inhibit motion artifacts during optical mapping.

### Experimental protocol of optical mapping

A pair of hook bipolar electrodes was inserted into the bottom of the heart for pacing. A pseudo-electrocardiograph was obtained with widely spaced bipolar electrodes to determine the ventricular rhythm. The ventricles were initially paced at a constant pacing cycle length (PCL) of 200 ms. The PCLs were progressively shortened (200, 150, 120, and 100 ms) with a duration of 3 s to record Vm and [Ca_i_] mapping. Pacing was performed with S1–S2 using a basic PCL of 100 ms and with S2 decreased by 2 ms until the ventricular effective refractory period was reached. Finally, optical recordings were then performed during ventricular tachycardia (VT)/VF inducibility.

### Statistical analysis

Data are expressed as mean ± SEM. Statistical analysis was performed using an unpaired Student’s *t*-test or one-way analysis of variance (ANOVA), followed by Tukey’s post hoc analysis. *p* < 0.05 was considered statistically different.

## Results

### Effects of empagliflozin on QT interval and ventricular arrhythmias in infarcted hearts of mice

**FIGURE 1:**
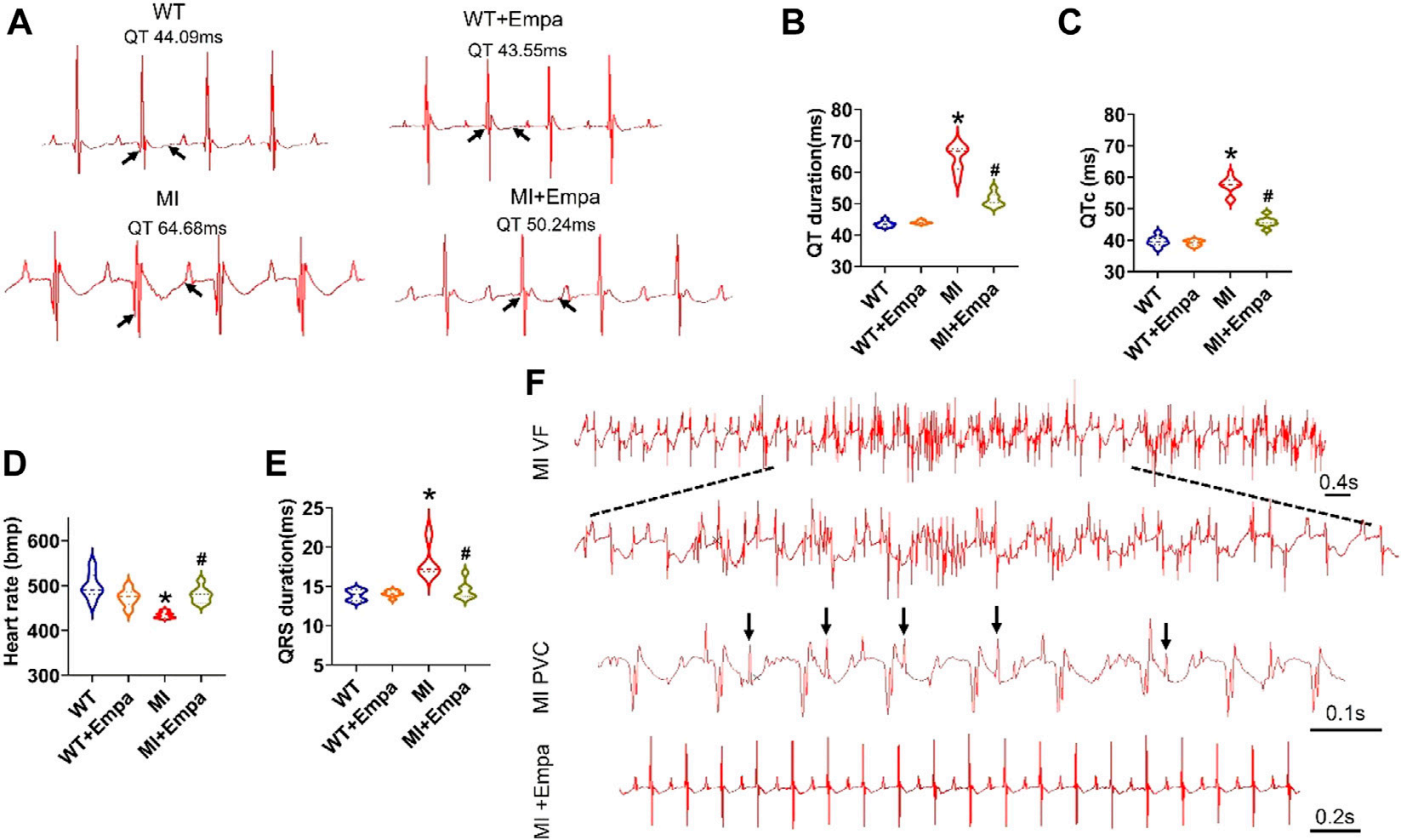
Effects of EMPA on QT interval and ventricular arrhythmias in chronic MI mice. **(A,B)** Representative ECG traces of the QT interval in WT, WT + EMPA, MI, and MI + EMPA. N = 6–10. *p < 0.05 vs*.* WT; ^#^ *p* < 0.05 vs*.* MI. *p*-values were determined using the unpaired *t* test. **(C)** QTc of WT, WT + EMPA, MI, and MI + EMPA mice. N = WT: 8; WT + EMPA: 6; MI: 10; MI + EMPA: 7; *p < 0.05 vs*.* WT; and ^#^ *p* < 0.05 vs*.* MI. **(D,E)** heart rate and QRS of WT, WT + EMPA, MI, and MI + EMPA mice. N = 6–9; *p < 0.05 vs*.* WT; and ^#^ *p* < 0.05 vs*.* MI. **(F)** Representative ECG traces of the VF and PVCs in MI and MI + EMPA mice.

A surface ECG was measured to determine the QT interval from anesthetized WT, WT + EMPA, MI, and MI + EMPA mice. In infarcted hearts, EMPA treatment significantly shortened the QT interval (MI vs. MI + EMPA: 64.68 vs. 50.24 ms; *p* < 0.05) (Figures 1A and B) and QTc interval (MI vs. MI + EMPA: 57.39 vs. 45.80 ms; *p* < 0.05) (Figure 1C). The QT intervals were not changed by EMPA in sham control (WT) mice. EMPA significantly increased the heart rate of MI hearts (MI vs. MI + EMPA: 434.98 bpm vs. 479.84 bpm; *p* < 0.05), while there was no change of heart rate in WT mice (Figure 1D). Furthermore, QRS intervals were shortened in MI + EMPA mice compared to MI (MI vs. MI + EMPA: 18.20 vs. 14.48 ms; *p* < 0.05) (Figure 1D). EMPA treatment led to reduced development of PVBs and VF in MI mice (Figure 1F).

### Effects of empagliflozin on action potential duration and Δaction potential duration of perfused infarcted hearts of mice

**FIGURE 2:**
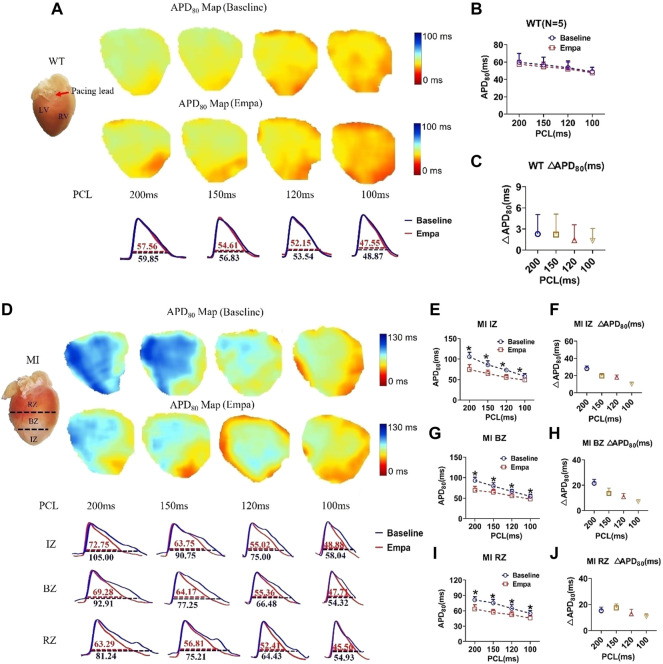
Effects of EMPA on action potential duration (APD) at different pacing cycle lengths (PCLs) in normal and infarcted hearts. **(A)** Representative membrane potential traces and APD at 80% repolarization (APD_80_) maps at baseline and in the presence of EMPA (10 nmol/L) in normal ventricles. **(B,C)** APD_80_ and ΔAPD_80_ associated with different PCLs at baseline and after EMPA infusion in normal ventricles. *N* = 5. **(D)** Representative membrane potential traces and APD at 80% repolarization (APD_80_) maps at baseline and in the presence of EMPA (10 nmol/L) in IZ, BZ, and RZ zones of infarcted hearts **(E,F)** APD_80_ and ΔAPD_80_ associated with different PCLs at baseline and after EMPA infusion in infarcted ventricles of IZ. *N* = 9–12. *p < 0.05 vs*.* baseline, *p*-values were determined by unpaired *t* test. **(G,H)** APD_80_ and ΔAPD_80_ associated with different PCLs at baseline and after EMPA infusion in infarcted ventricles of BZ. *N* = 10–12. *p < 0.05 vs*.* baseline. **(I,J)** APD_80_ and ΔAPD_80_ associated with different PCLs at baseline and after EMPA infusion in infarcted ventricles of RZ. *N* = 9–11. *p < 0.05 vs*.* baseline.

We further evaluated the effects of EMPA on APD of *ex vivo* perfused MI hearts by optical mapping. At baseline, the mean APD at 80% repolarization (APD_80_) in the IZ, BZ, and RZ of MI ventricles was longer than that in the normal ventricles at all PCLs (Figures 2A,D). EMPA treatment did not produce a significant influence on APD_80_ in normal ventricles, and the ΔAPD_80_ is very small (Figures 2B,C). In the infarcted ventricles, EMPA shortened APD_80_ at all pacing cycle lengths (PCLs) in IZ, BZ, and RZ; however, the effects were more apparent at longer PCLs, i.e., in the IZ of infarcted ventricles, at PCL200 ms, baseline: 105 ms, EMPA: 72.75 ms, *p* < 0.05; at PCL 150 ms, baseline: 90.75 ms, EMPA: 63.75 ms, *p* < 0.05 (Figures 2D,E); in the BZ of infarcted ventricles, at PCL200 ms, baseline:92.91 ms, EMPA: 69.28 ms, *p* < 0.05; at PCL 150 ms, baseline: 77.25 ms, EMPA: 64.14 ms, *p* < 0.05 (Figures 2D,G); and in the RZ of infarcted ventricles, at PCL200 ms, baseline: 81.24 ms, EMPA: 63.29 ms, *p* < 0.05; at PCL 150 ms, baseline: 75.21 ms, EMPA: 56.81 ms, *p* < 0.05; *p* < 0.05, Figures 2D,I). EMPA also shortened ΔAPD_80_ at all pacing cycle lengths (PCLs) in the infarcted ventricles IZ, BZ, and RZ (Figures 2F,H,J).

### Effects of empagliflozin on conduction velocity and AP rise time of perfused infarcted hearts of mice

**FIGURE 3:**
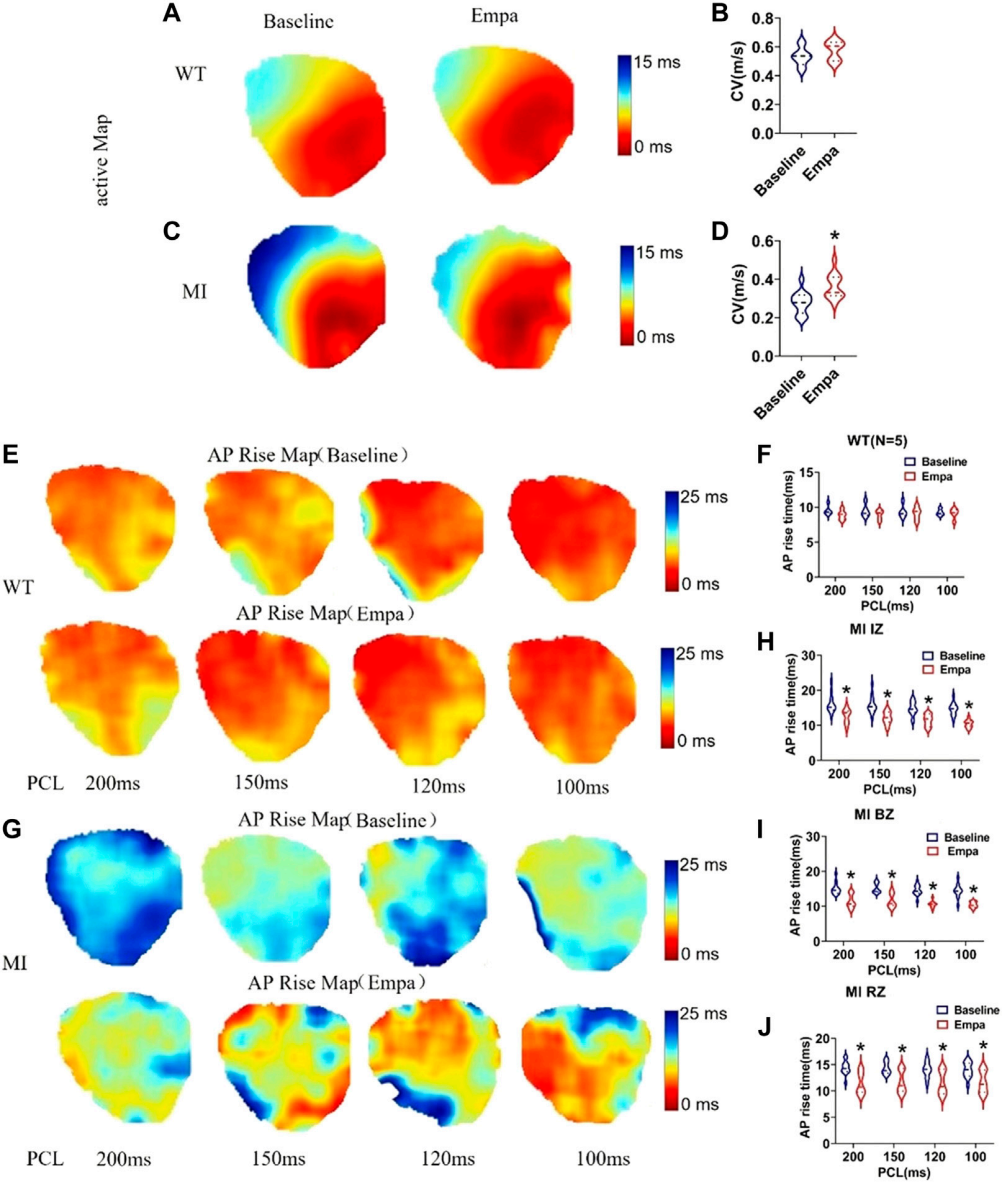
Effects of EMPA on conduction velocity (CV) and AP rise time at different pacing cycle lengths (PCLs) in normal and infarcted ventricles. **(A,C)** Maps of activation at baseline and in the presence of EMPA (10 umol/L) in normal and infarcted ventricles. **(B,D)** conduction velocity at baseline and after EMPA infusion in normal and infarcted ventricles. *N* = 5 for WT; *N* = 13 for MI. *p < 0.05 versus baseline. *p*-values were determined using the unpaired *t* test. **(E)** Action potential (AP) rise time (TRise) maps associated with different PCLs at baseline and in the presence of EMPA (10 umol/L) in normal ventricles. **(F)** TRise associated with different PCLs at baseline and after EMPA infusion in normal ventricles. *N* = 5_._ **(G)** TRise maps associated with different PCLs at baseline and in the presence of EMPA in infarcted ventricles. **(H–J)** TRise associated with different PCLs at baseline and after EMPA infusion in infarcted ventricles of IZ, BZ, and RZ. *N* = 11–13_._ *p < 0.05 versus baseline. *p*-values were determined using the unpaired *t* test.

In the infarcted ventricles, the conduction velocity map showed that EMPA treatment did not significantly change conduction velocity in normal ventricles (Figures 3A,B), while it significantly increased the conduction velocity of infarcted hearts (Figures 3C,D). AP rise maps at baseline and after EMPA infusion did not change in all PCLs from recordings in the WT mice hearts (Figures 3E,F). In the infarcted ventricles, EMPA shortened AP rise time at all PCLs in IZ, BZ, and RZ zones, and the effects were more apparent at longer PCLs (Figures 3G–J).

### Effects of empagliflozin on CaT duration and ΔCaT of perfused infarcted hearts of mice

**FIGURE 4:**
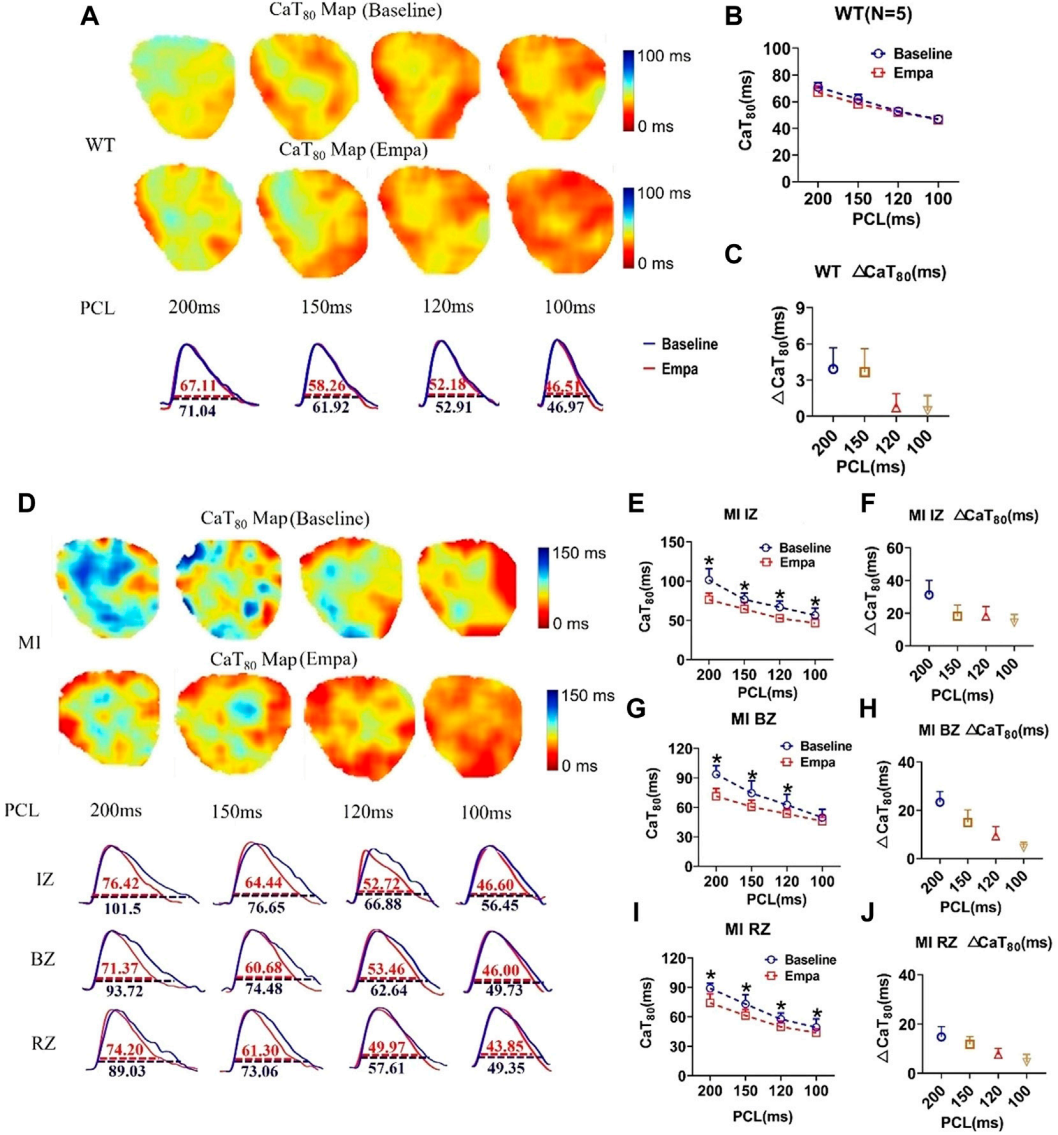
Effects of EMPA on Ca^2+^ transient duration (CaTD) at different pacing cycle lengths (PCLs) in normal and infarcted ventricles. **(A)** Representative Ca^2+^ transient traces and CaT at 80% repolarization (CaT_80_) maps at baseline and in the presence of EMPA (10 umol/L) in normal ventricles. **(B,C)** CaT_80_ and ΔCaT_80_ associated with different PCLs at baseline and after EMPA infusion in normal ventricles. *N* = 5. **(D)** Representative Ca^2+^ transient traces and CaT_80_ maps at baseline and in the presence of EMPA in infarcted ventricles of IZ, BZ, and RZ. **(E,F)** CaT_80_ and ΔCaT_80_ associated with different PCLs at baseline and after EMPA infusion in infarcted ventricles of IZ. *N* = 9. *p < 0.05 vs*.* baseline, *p*-values were determined by unpaired *t* test. **(G,H)** CaT_80_ and ΔCaT_80_ associated with different PCLs at baseline and after EMPA infusion in infarcted ventricles of BZ. *N* = 10–11. *p < 0.05 vs*.* baseline. **(I,J)** CaT_80_ and ΔCaT_80_ associated with different PCLs at baseline and after EMPA infusion in infarcted ventricles of RZ. *N* = 9. *p < 0.05 vs*.* baseline.

We investigated the effects of EMPA on Ca^2+^ cycling characteristics by comparing the 80% Ca^2+^ transient duration (CaTD_80_). CaT_80_ maps at baseline and after EMPA infusion were not changed in the hearts of WT mice. EMPA did not significantly change CaT_80_ in normal ventricles, and ΔCaT_80_ is very small (Figures 4A–C). In the infarcted ventricles, EMPA shortened CaT_80_ at all PCLs in IZ, BZ, and RZ zones, and the effects were more apparent at longer PCLs (Figures 4D,E,J,I). EMPA also shortened ΔCaT_80_ at all PCLs in the IZ, BZ, and RZ zones of infarcted ventricles (Figures 4F,H,J).

### Effects of empagliflozin on CaT rise time and [Ca_i_] transient decay constant Tau of perfused infarcted hearts of mice

**FIGURE 5:**
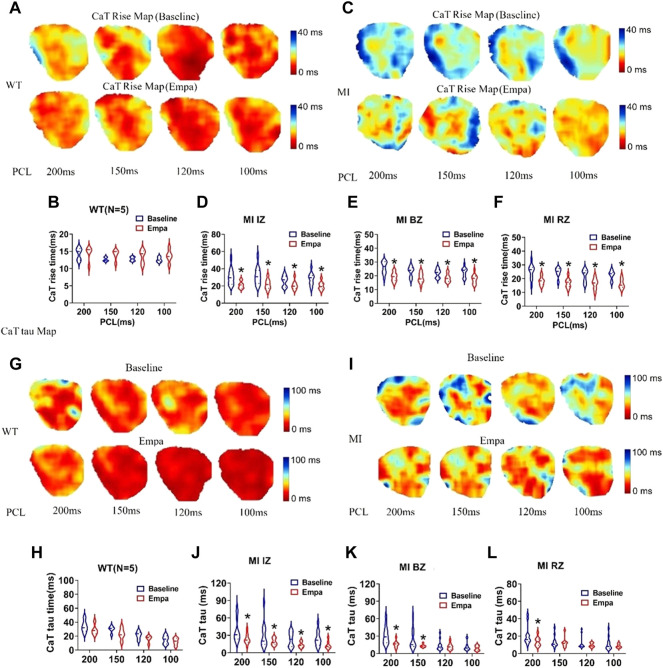
Effects of EMPA on Ca^2+^ transient rise time (CaTRise) and Tau at different pacing cycle lengths (PCLs) in normal and infarcted ventricles. **(A)** Ca^2+^ transient rise time (CaTRise) maps associated with different PCLs at baseline and in the presence of EMPA (10 umol/L) in normal ventricles. **(B)** CaTRise associated with different PCLs at baseline and after EMPA infusion in normal ventricles. *N* = 5_._ **(C)** CaTRise maps associated with different PCLs at baseline and in the presence of EMPA in infarcted ventricles. **(D–F)** CaTRise associated with different PCLs at baseline and after EMPA infusion in infarcted ventricles of IZ, BZ, and RZ. *N* = 9–12. *p < 0.05 versus baseline. *p*-values were determined using an unpaired *t* test. **(G,H)** Ca^2+^ transient decay time (Tau) maps and Tau associated with different PCLs at baseline and in the presence of EMPA normal ventricles. *N* = 5. **(I–L)** Tau maps and Tau associated with different PCLs at baseline and in the presence of EMPA in infarcted ventricles of IZ, BZ, and RZ. *N* = 10–13_._ *p < 0.05 versus baseline. *p*-values were determined using an unpaired *t* test.

In the normal ventricles, the CaT rise map did not change at baseline and after EMPA infusion at all PCLs (Figures 5A,B). However, in the infarcted ventricles, after EMPA infusion, the CaT rise time significantly shortened at all PCLs in IZ, BZ, and RZ zones of MI hearts (Figures 5C–F). [Ca_i_] transient decay constant Tau maps were not changed in all PCLs at baseline and after EMPA infusion in the hearts of WT mice (Figures 5G,H). In infarcted ventricles, EMPA decreased Tau at all PCLs in IZ, BZ, and RZ zones (Figures 5I–L).

### Secondary rises of [Ca_i_] and early afterdepolarization in infarcted ventricles were improved by EMPA

**FIGURE 6:**
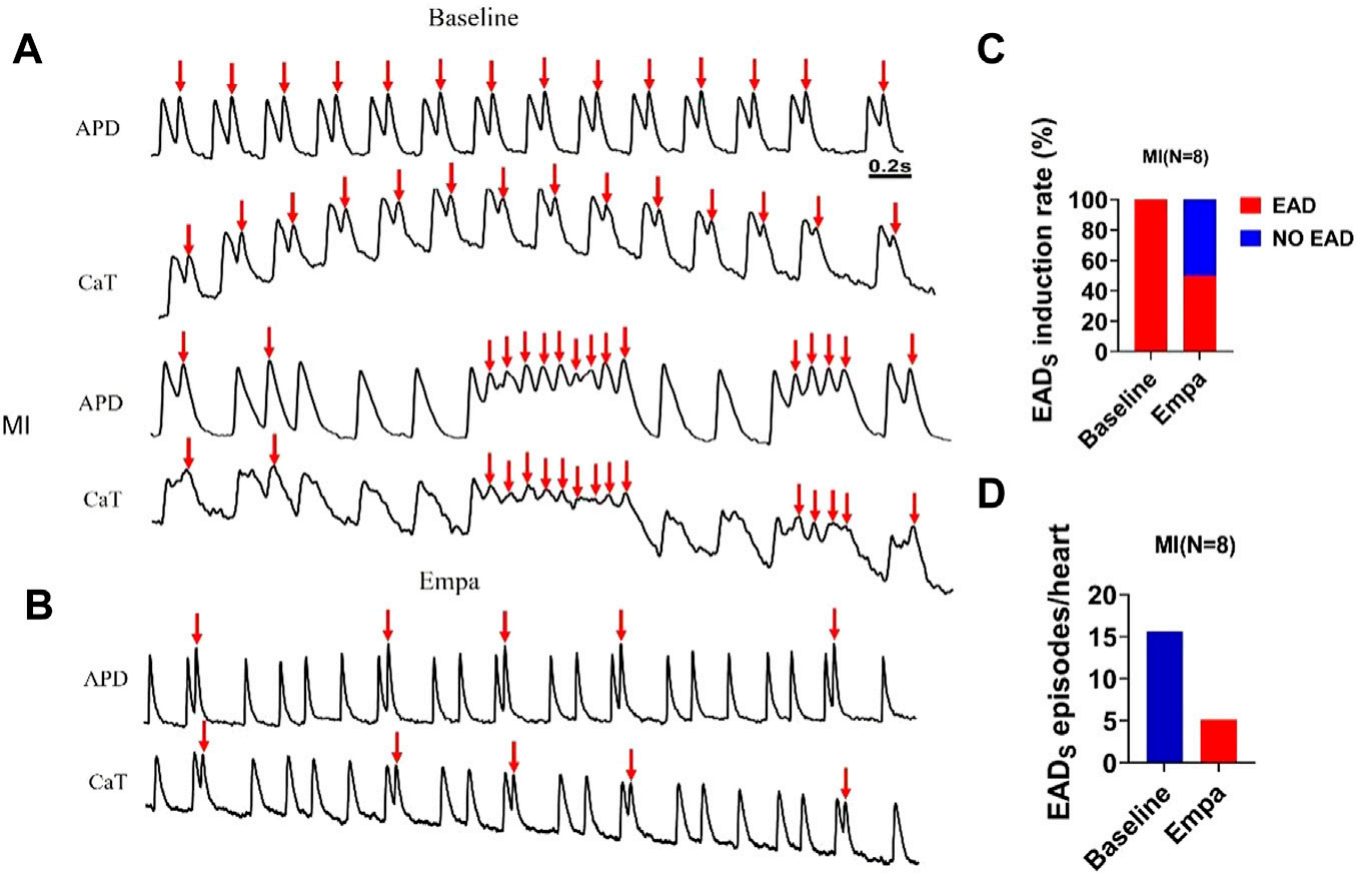
EMPA reduced secondary rises of [Ca_i_] and EADs in infarcted ventricles. **(A,B)** EAD and Secondary rises of [Ca_i_] traces at baseline and in the presence of EMPA in infarcted ventricles. **(C,D)** EAD induction rate and EADs episodes at baseline and in the presence of EMPA. *N* = 8.

We observed a secondary rise of [Ca_i_] in infarcted ventricles both at baseline and after EMPA administration (Figures 6A,B). The secondary rise of [Ca_i_] was decreased in the presence of EMPA, along with reduced EADs (red arrows at site). At baseline, the spontaneous secondary rise of [Ca_i_] was detected, and EADs was increased in infarcted ventricles. The activation sites of EADs beats colocalized with the highest secondary rises of [Ca_i_] regions. We analyzed the total EADs episodes in infarcted hearts and found that it was inhibited by EMPA (baseline: 15.625, EMPA: 5.125 episodes per heart). The activation site of EADs always occurred at the site with longer APD and larger amplitude of the secondary rises of [Ca_i_]. EMPA administration reduced secondary [Ca_i_] rises, [Ca_i_] transient duration, EAD induction rate, and EAD episodes (Figures 6C,D).

### Empagliflozin reduces the ventricular fibrillation vulnerability in infarcted ventricles

**FIGURE 7:**
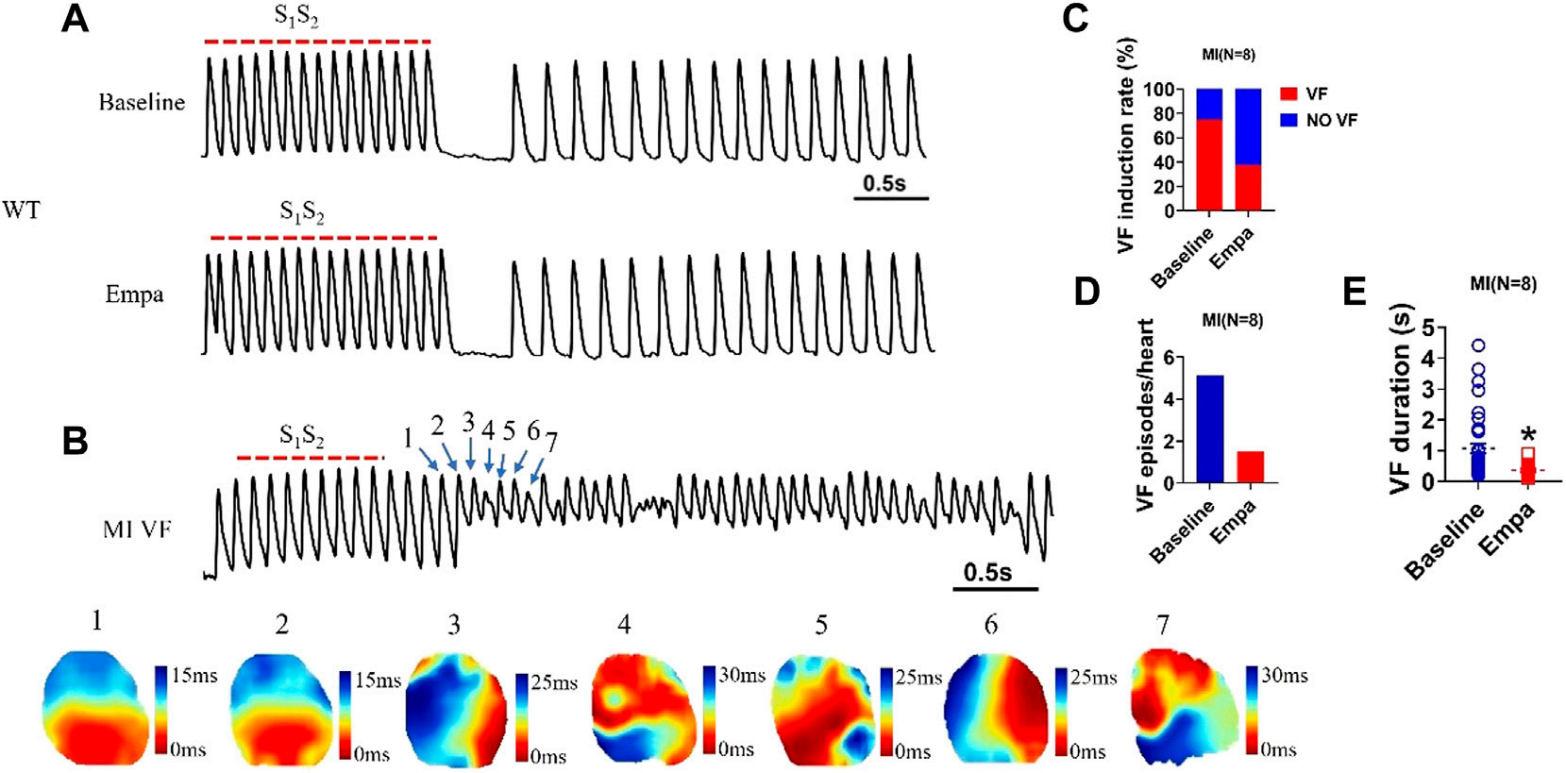
EMPA reduced the VF vulnerability in infarcted ventricles. **(A)** Ventricular fibrillation (VF) induced by S1S2 pacing at baseline and in the presence of EMPA in normal ventricles. *N* = 5 mice. **(B)** Ventricular fibrillation (VF) and active map by S1S2 pacing at baseline in infarcted ventricles. *N* = 8 mice. **(C–E)** VF induction rate and duration at baseline and in the presence of EMPA in infarcted ventricles. *N* = 8 mice. *p < 0.05 versus baseline. *p*-values were determined using an unpaired *t* test.

VF was rarely induced in the normal ventricles at a PCL of 100 ms at baseline or after EMPA with an S1/S2 pacing protocol (Figure 7A). In infarcted ventricles, at baseline, there was significantly induced VF at a PCL of 100 ms. We performed a specific focus on how VFs are initiated in the infarcted ventricles. An example of VF induced with an S1/S2 pacing protocol is shown in Figure 7B Active maps of the 1–7 wave showed the earliest activation of the VF occurred at the sites. After EMPA infusion, either VF induction rate, VF episodes, or VF duration was decreased in infarcted ventricles (Figures 7C–E).

### Effect of empagliflozin on ventricular fibrillation dynamics in infarcted ventricles

**FIGURE 8:**
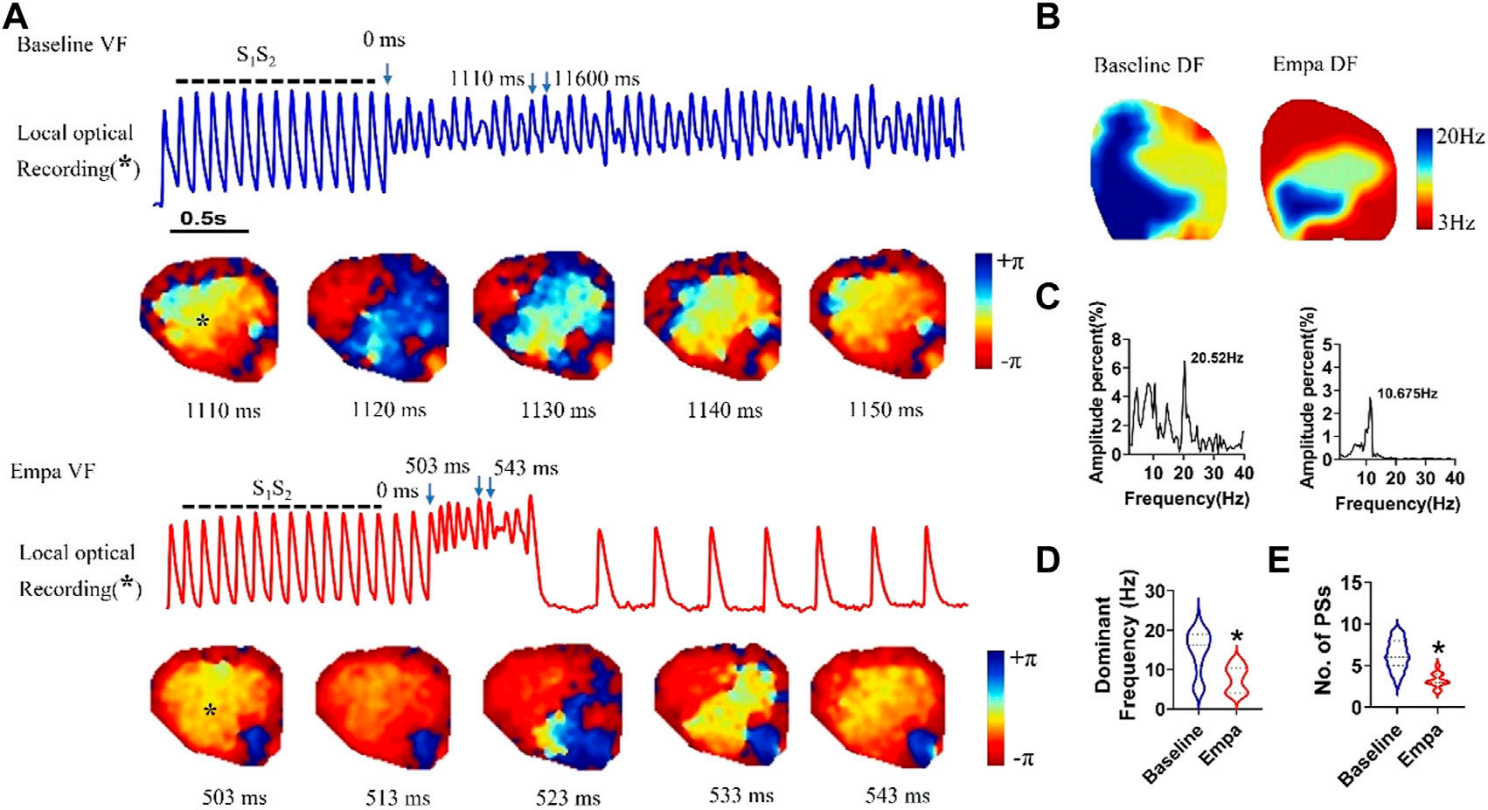
Effects of EMPA on the wave breaks and dominant frequency of ventricular fibrillation in infarcted ventricles. **(A)** Recordings of S1S2 pacing-induced sustained VF episodes at baseline and after EMPA infusion in an infarcted ventricle. Top, corresponding optical recording of VF at the asterisk site. Consecutive phase maps sampled at 10 ms intervals during VF at baseline and after EMPA infusion. **(B–D)** DF distribution of VF at baseline and after EMPA infusion. *N* = 10 mice. *p < 0.05 versus baseline. **(E)** Effects of EMPA on the number of phase singularities before and after EMPA infusion in infarcted ventricles. Note that the numbers of phase singularity are decreased by EMPA. *N* = 10 mice. *p < 0.05 versus baseline. *p*-values were determined using an unpaired *t* test.

We analyzed the VF dynamics in infarcted ventricles, and consecutive phase maps sampled at 10 ms intervals during VF were analyzed for phase singularities (PSs). EMPA decreased the number of phase singularities compare to baseline (*p* < 0.05; Figures 8A,E). The dominant frequency of VF was decreased from 20.52 Hz at baseline to 10.675 Hz after EMPA infusion (*p* < 0.05; Figures 7B–D).

## Discussion

**FIGURE 9:**
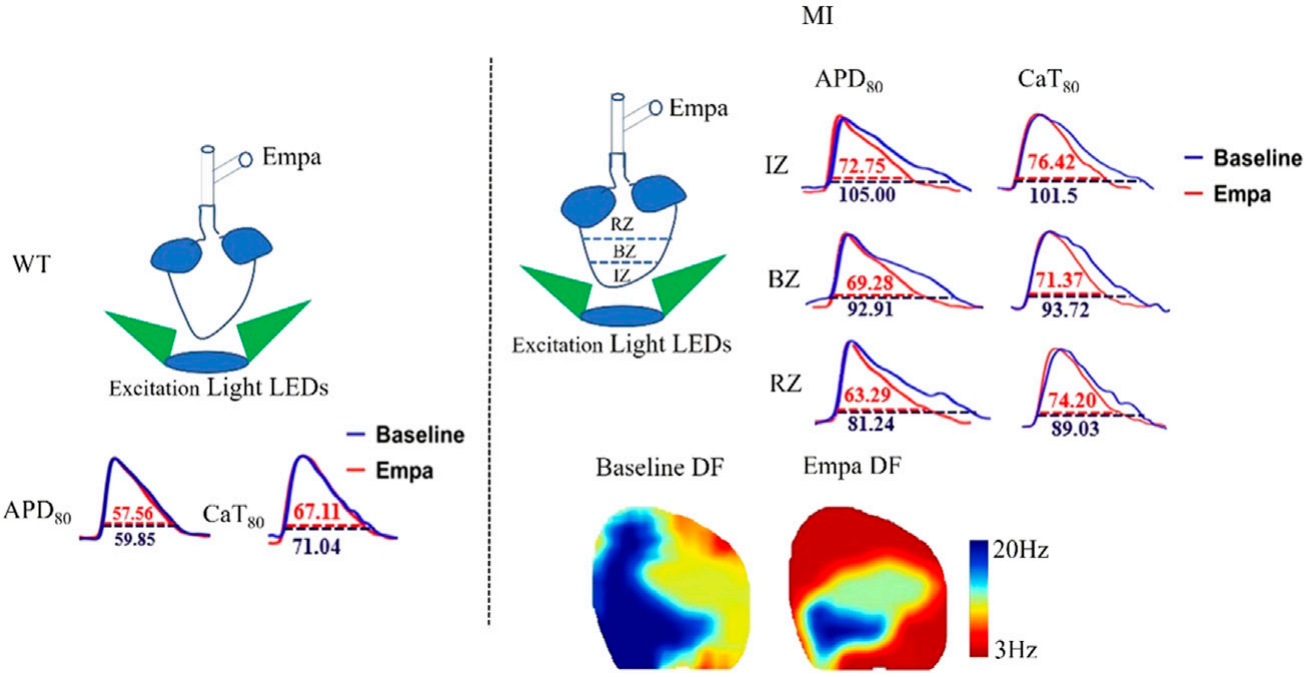
Schematic summary of the signaling pathway of EMPA and arrhythmia in post-MI.

In this paper, we identified the following key observations (Figure 9): 1) EMPA inhibited the prolongation of QT interval and induction of ventricular arrhythmias in infarcted ventricles; 2) the APD_80_ and AP rise time were shortened by EMPA in the IZ, BZ, and RZ zones of infarcted ventricles; 3) the CaT_80_, CaT rise time, and Tau were shortened by EMPA in the IZ, BZ, and RZ zones of infarcted ventricles; 4) EMPA reversibly reduced the incidence of calcium disturbances-induced EADs and VF in infarcted ventricles; and 5) EMPA prevented activation of the PSs and DF and improved functional recovery in post-MI hearts.

The role of SGLT isoforms in myocardial physiology is controversial (Di Franco et al., 2017; Ng et al., 2018). However, studies have shown that SGLT1 is abundantly expressed in the heart, whereas SGLT2 is barely detectable (Banerjee et al., 2009; Li et al., 2019b; Kondo et al., 2021). SGLT2 inhibitors improve heart failure process independent of hypoglycemic mechanisms (Raz and Cahn, 2016; Shah and Fang, 2022). Empagliflozin is the most selective SGLT2 inhibitor (Anker and Butler, 2018), it suppresses cardiomyocyte autophagic cell death to confer cardioprotective effects. In myocardial infarction (MI) mouse models with and without diabetes mellitus, EMPA treatment significantly reduced the infarct size and myocardial fibrosis (Jiang et al., 2022). In addition to participating in the process of heart failure, EMPA also plays a role in cardiac arrhythmia. EMPA reduced late-*I*_Na_ in cardiomyocytes from mice with heart failure and cardiac Nav1.5 sodium channels containing the long QT syndrome 3 mutations R1623Q or ΔKPQ (Philippaert et al., 2021). In this study, we demonstrated the effect of EMPA on arrhythmia of a chronic MI mouse model.

Induction of arrhythmias after myocardial infarction is associated with an increased risk of sudden death (Zaman and Kovoor, 2014; Chatterjee and Levy, 2020). Following MI, the risk for ventricular tachyarrhythmia and sudden cardiac death in patients is increased (Hegyi et al., 2018; Li et al., 2020; Liu et al., 2021). QT interval and changes in the corrected QT (QTc) were important indicators of APD, which is mainly determined by ventricular repolarization. QT interval and QTc were significantly increased after MI (Tao et al., 2020). We discovered that QT and QTc were obviously recovered after EMPA treatment in post-MI mice. In chronic ischemic cardiomyopathy, VF is most often attributable to APD repolarization heterogeneity and conduction abnormalities in the IZ, BZ, and RZ zones (Saffitz and Kleber, 2012; Arbustini et al., 2018). We found that acute perfusion of EMPA shortened APD_80_ in the infarcted ventricles. This indicates that EMPA may transiently affect channel currents rather than channel proteins. EMPA was shown to directly decrease late-*I*_Na_ current in the mouse heart failure model (Philippaert et al., 2021). In this study, we found EMPA increased conduction velocity and reduced the rise time of action potential, which may be related to its influence on the sodium channel in infarcted ventricles. The Ca^2+^ influx triggers Ca^2+^ release by activating ryanodine receptors (RyRs) in the SR membrane from intracellular sarcoplasmic reticulum (SR) Ca^2+^ stores (Shen, 2006; Yang et al., 2020). Resting calcium may increase when [Ca_i_] is taken up again into the SR by the sarcoplasmic/endoplasmic reticulum Ca^2+^ ATPase (SERCA), and extrusion from the cell primarily via sarcolemmal sodium–calcium exchange (NCX) was suppressed (Zhang et al., 2018; Gorski et al., 2019). The contractile dysfunction occurring in MI or hypoxia was primarily caused by impairment of intracellular Ca^2+^ homeostasis due to the disturbance of Ca^2+^ handling (McCrink et al., 2017; Zhang et al., 2018). The known Ca^2+^ handling dysregulation in MI includes calcium transient duration increase (Maczewski and Mackiewicz, 2008), intracellular Ca^2+^ overload (Morciano et al., 2015), and Ca^2+^ leak via RYR_2_ channels from SR (Valverde et al., 2019). In infarcted hearts, EMPA reduced CaT_80_ duration, increased CaT rise time, and shortened calcium transient decay constant Tau, indicating that EMPA improved calcium cycling during MI. The secondary rise of [Ca_i_] during late action potential plateau is commonly observed in MI but not in normal hearts (Chang et al., 2015). The mechanism of the secondary rise of [Ca_i_] is attributed to Ca^2+^ entry increase, which further promotes greater Ca^2+^ entry and additional sarcoplasmic reticulum Ca^2+^ release during phases 2 and 3 of the action potential (Chang et al., 2013; Chang et al., 2018). Under these conditions, EAD take-off potentials were observed to occur in action potential repolarization, suggesting a secondary rise of [Ca_i_], and calcium cycling contributes to EAD formation, possibly including other ionic mechanisms. Our data demonstrated that EMPA reduced the secondary rise of [Ca_i_] numbers and triggers; decreased EADs, PVBs, and VF occurrence; and improved the PS and DF in infarcted ventricles.

In conclusion, EMPA is a novel regulator of APD and calcium transient remodeling after chronic MI, which suppresses the prolongation of APD, the disturbance of calcium handling, and reduced arrhythmia susceptibility. The current study enriched our understanding of the electrophysiological action of SGLT2 inhibitors and indicated that EMPA possesses the potential to become therapeutic agents for ventricular arrhythmias.

## Data Availability

The raw data supporting the conclusions of this article will be made available by the authors without undue reservation.
